# Identification of stacking faults in silicon carbide by polarization-resolved second harmonic generation microscopy

**DOI:** 10.1038/s41598-017-05010-y

**Published:** 2017-07-07

**Authors:** Radu Hristu, Stefan G. Stanciu, Denis E. Tranca, Efstathios K. Polychroniadis, George A. Stanciu

**Affiliations:** 1 Center for Microscopy-Microanalysis and Information Processing, University Politehnica of Bucharest, 313 Splaiul Independentei, 060042 Bucharest, Romania; 2 Physics Department, Aristotle University of Thessaloniki, 54124 Thessaloniki, Greece

## Abstract

Although silicon carbide is a highly promising crystalline material for a wide range of electronic devices, extended and point defects which perturb the lattice periodicity hold deep implications with respect to device reliability. There is thus a great need for developing new methods that can detect silicon carbide defects which are detrimental to device functionality. Our experiment demonstrates that polarization-resolved second harmonic generation microscopy can extend the efficiency of the “optical signature” concept as an all-optical rapid and non-destructive set of investigation methods for the differentiation between hexagonal and cubic stacking faults in silicon carbide. This technique can be used for fast and *in situ* characterization and optimization of growth conditions for epilayers of silicon carbide and similar materials.

## Introduction

Silicon carbide (SiC) represents a class of wide-bandgap semiconductors existing in a large variety of crystal structures known as polytypes, which are a one dimensional disorder allowing for the formation of structures with different stacking sequences. Polytypes are named by the shape of the unit cell (cubic, hexagonal, rhombohedral) and the number required to establish a unit cell. The polytypes that are currently most commonly used for device research are two hexagonal species 4H- and 6H-SiC and the cubic polytype 3C-SiC.

Being a highly promising crystalline material for high-temperature, high-frequency and high-power devices^1^, SiC and in particular 4H-SiC were successfully used to date in the development of a wide range of electronic devices e.g. field-effect transistors^2, 3^ and bipolar transistors^4^. However, similar to the case of many other semiconductors, SiC structures may contain both extended and point defects that can perturb the lattice periodicity. Such defects hold deep implications with respect to device reliability. Despite significant improvements made in SiC crystal growth techniques during the past years the number of crystallographic defects still remains an important problem. It was shown that extended defects like stacking faults (SFs)^5–7^, micropipes^8^, screw dislocations and basal plane dislocations^9^ play a critical role in the performance degradation and reliability of SiC power devices^10, 11^. Although numerous studies on the formation and propagation of defects during crystal growth have been carried out, the mechanisms behind SiC extended defects is not yet fully understood. There is thus a great need for developing new methods that can detect and reduce SiC defects which are detrimental to device functionality and to apply these in an industrial relevant manner for improving SiC crystal manufacturing technologies.

In the case of 4H-SiC one of the most common defects are SFs which are local regions of incorrect stacking of crystallographic planes associated with the presence of partial dislocations. Different types of SFs have been theoretically investigated to increase the understanding of their presence and formation. In such previous studies addressing SFs formation and implications, it was shown that Shockley SFs in a hexagonal SiC matrix lead to a narrow band of 3C polytype^12^, forming cubic SiC regions of variable thicknesses embedded in the 4H-SiC epilayer, while 8H-like structures have also been reported^6^.

To date‚ various imaging techniques have been employed to identify, determine the density and characterize different defects in SiC. Among these one can find both destructive techniques such as chemical etching of the surface^13^, transmission electron microscopy (TEM)^14, 15^ and non-destructive techniques: e.g. synchrotron white-beam X-ray topography^12^, atomic force microscopy (AFM)^16^, luminescence-based techniques^17^ such as cathodoluminescene^18^, electroluminescence^7, 19^ and photoluminescence^20–22^. Additional optical non-destructive techniques are based on second harmonic generation (SHG) microscopy which has been demonstrated as a powerful tool for probing the symmetry of materials. SHG microscopy has been used for polytype identification^23, 24^ and for the detection and analysis of extended structural defects in SiC epilayers^20, 25^. SHG microscopy is not limited to intensity measurements, and using for example the coherent nature of SHG, polarization-resolved SHG (PSHG) microscopy can provide additional information about the arrangement and the crystalline structure of the sample with extended applications in biophotonics for the characterization of collagen organization^26–28^, but also in materials sciences^25, 29, 30^. In order to evaluate the nonlinear optical response of the sample‚ the anisotropy factor (β) was previously used to assess the orientation differences of the potassium dihydrogen phosphate micro-crystals^29^, a standard nonlinear optical crystal. Until now, by using only optical investigation techniques, the concept of SFs “optical signature” was introduced^17^ for the non-destructive and rapid classification of SFs in SiC. This “optical signature” combines results from panchromatic room temperature chatodoluminescence with low temperature photoluminescence to classify different types of SFs in 4H-SiC. The main limitation of this method stands in its weak sensitivity to the polytype when changing from 3C to 8H in 4H-SiC.

In this study, we show that by combining PSHG imaging and the analysis of the SHG rotational anisotropy by means of the anisotropy factor can lead to the identification of SFs in SiC epilayers. We demonstrate that PSHG and the anisotropy factor can be used for the differentiation of the 3C and 8H behavior of SFs in 4H-SiC epilayers, which promises to be an important application in the characterization and optimization of growth conditions for epilayers of SiC and similar materials.

## Results

**Figure 1:**
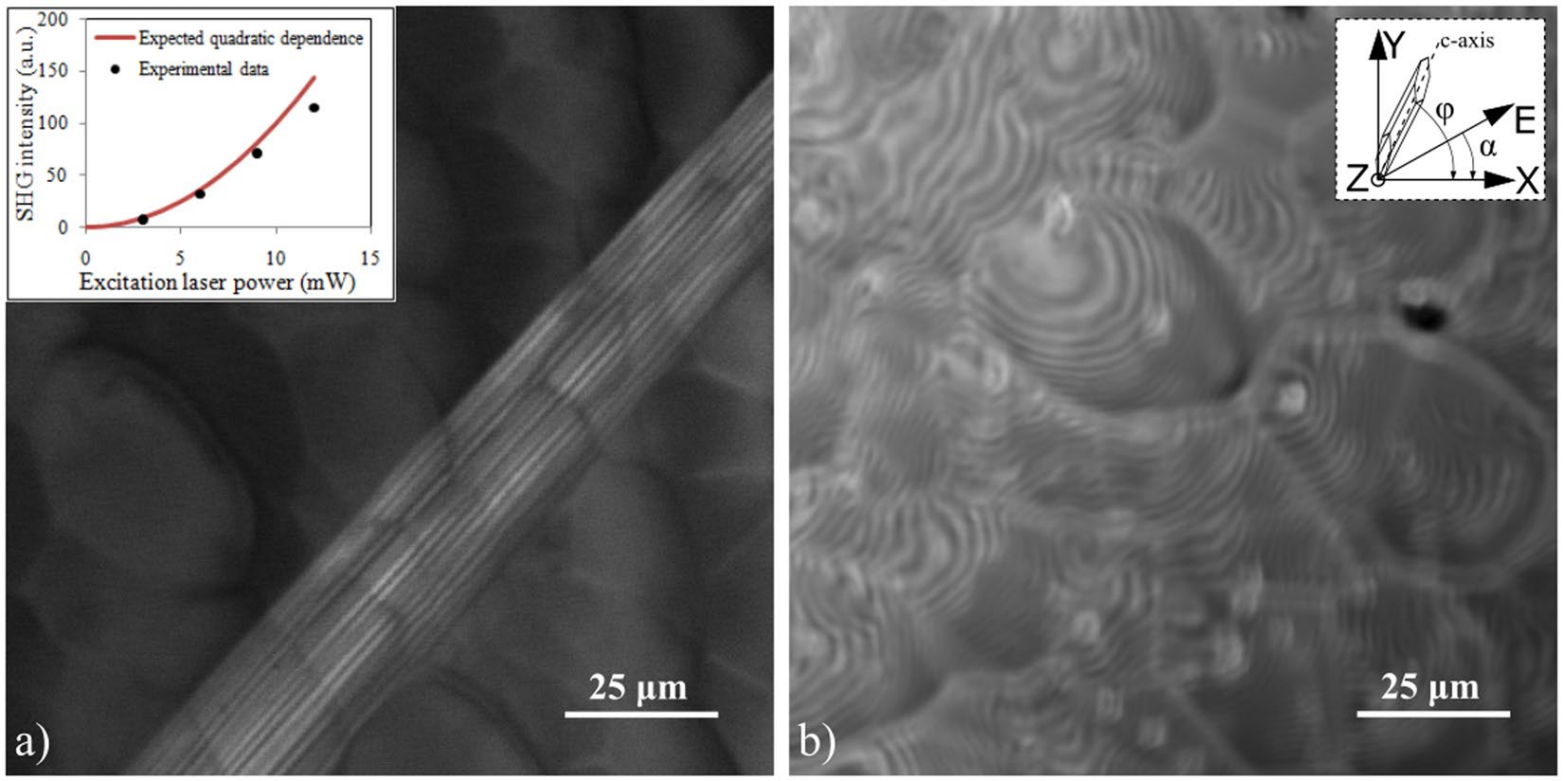
Images of the 4H-SiC sample proving that the SFs are only visible in the nonlinear optical image. (**a**) The polarization-independent SHG image obtained by using a circular polarized laser beam (excitation laser power 10 mW). The inset shows the SHG intensity dependence on the excitation laser beam power. The solid line represents the expected quadratic dependence for the SHG process. (**b**) A confocal microscopy reflectance image of the same area as in Fig. 1(a) which contains no information on the SFs in the 4H-SiC sample. The inset reveals the significance of α (laser beam polarization angle) and φ (crystal structure orientation angle) when considering the coordinates of the image plane (x, y).

A typical SHG image of the SiC sample containing SFs is shown in Fig. 1 with the bright and dark areas representing areas with strong and weak SHG intensities, respectively. For comparison, an image of the same area taken by confocal laser scanning microscopy (CLSM) in reflectance mode is also shown in Fig. 1b. In the case of the investigated sample, the crystal structure of the SiC specimen contains an unusual number of defects. As can be observed in Fig. 1a, these are only visible in the SHG image and not in the CLSM one. A power dependence measurement was realized by adjusting the laser power from 3 mW to 12 mW (inset in Fig. 1a); these values were measured after the imaging objective (inset in Fig. 1a). The estimated SHG intensity was demonstrated to follow the square power dependence quite well proving that the recorded optical signals correspond to SHG. The values are slightly under the fitting curve at higher power values due to a saturation effect which appears on several pixels in the SHG images.

**Figure 2:**
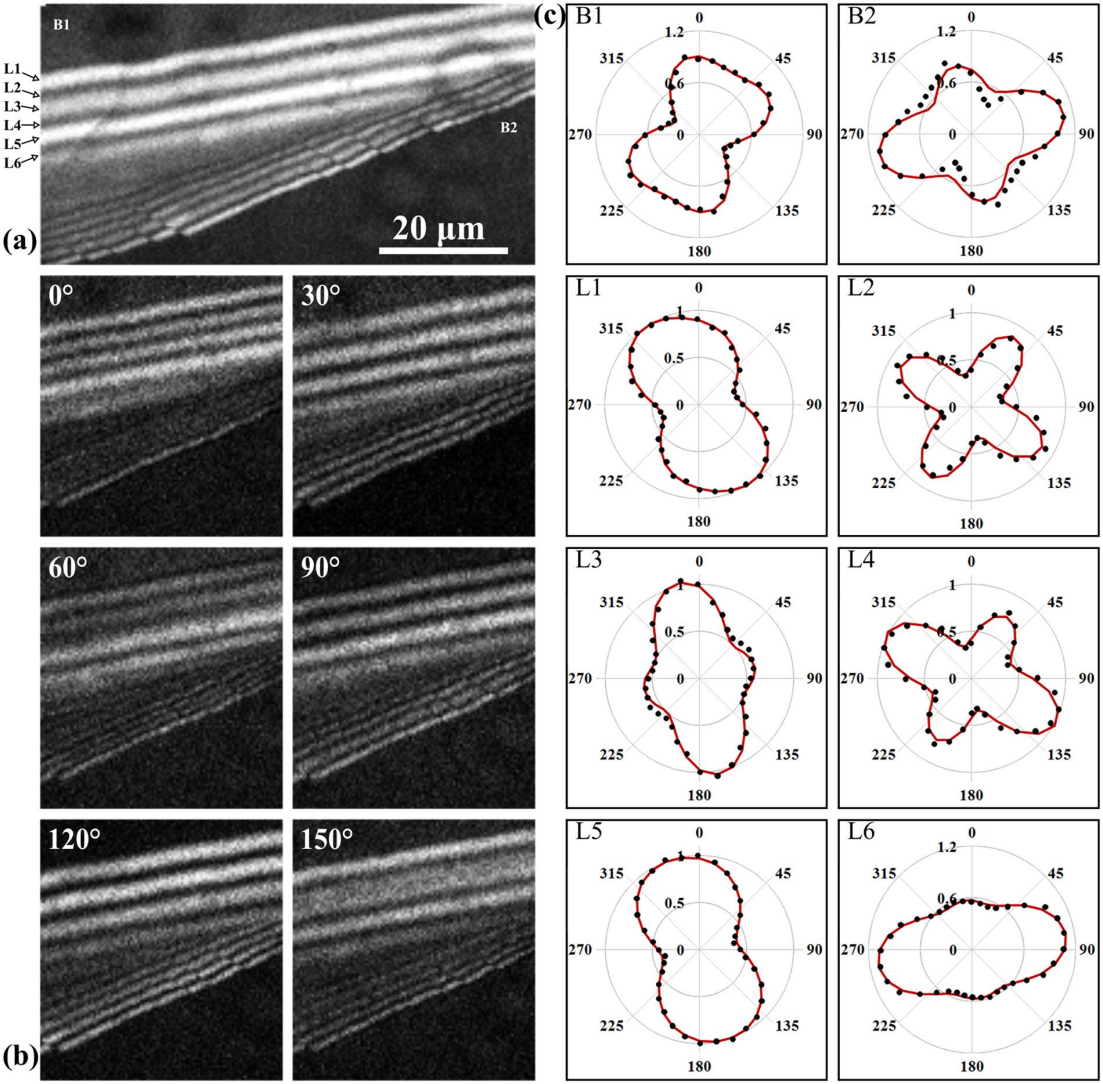
SHG intensity dependence on the laser beam polarization for a 4H-SiC epilayer containing SFs. (**a**) Polarization-independent SHG image obtained as the sum of all the images acquired with tunable linear laser beam polarization. The eight considered ROIs coded L1 to L6, B1 and B2 are illustrated. (**b**) PSHG images for six different linear laser beam polarizations. (**c**) The average SHG intensity as a function of the incident polarization angle α (varied from 0° to 360° in steps of 10°) for the two regions surrounding the defected area (B1 and B2) and the six alternating high and low SHG intensity lines (L1–L6) marked in (**a**). The black dots in the polar plots correspond to the experimental data points while the solid line is the fitting with the theoretical curve, either Eqs 1, 2 or a linear combination of the two.

As mentioned in the Introduction section, our experiment goes beyond simple intensity-based imaging by acquiring polarization-resolved SHG images (Fig. 2b) and computing the SHG intensity dependence on the incident laser beam polarization angle (Fig. 2c) which is varied from 0° to 360° in steps of 10°. In this case no analyzer is inserted before the detector. The parameter values computed by fitting the experimental data with the theoretical dependence (see Methods section) are listed in Table 1.

**Table 1:** Parameters retrieved by the fitting of the experimental data with either Eqs 1, 2 or a linear combination of the two. The behavior of each ROI is indicated (4H, 3C, 8H or a combination of 3C and 8H) as resulted from the fitting with the theoretical curves.

ROI	${\boldsymbol{-}}{{\boldsymbol{\chi }}}_{{\bf{33}}}^{{\boldsymbol{(}}2{\boldsymbol{)}}}/{{\boldsymbol{\chi }}}_{{\bf{15}}}^{{\boldsymbol{(}}2{\boldsymbol{)}}}$	${{\boldsymbol{\chi }}}_{{\bf{31}}}^{{\boldsymbol{(}}2{\boldsymbol{)}}}/{{\boldsymbol{\chi }}}_{{\bf{15}}}^{{\boldsymbol{(}}2{\boldsymbol{)}}}$	φ [°]	R²
B1 (4H)	1.606 ± 0.052	0.992 ± 0.084	29 ± 3.4	0.99
B2 (4H)	1.628 ± 0.06	1.344 ± 0.067	76.8 ± 5.2	0.81
L1 (3C & 8H)	1.895 ± 0.018	0.984 ± 0.107	71.8 ± 1.3 (3C) −21.7 ± 2.3 (8H)	0.99
L2 (3C)	—	—	77.8 ± 1.8	0.93
L3 (8H)	1.82 ± 0.094	1.076 ± 0.103	−12 ± 2.5	0.97
L4 (3C & 8H)	1.93 ± 0.026	0.948 ± 0.049	76.5 ± 1.4 (3C) −70.5 ± 5.4 (8H)	0.96
L5 (3C & 8H)	1.903 ± 0.014	0.994 ± 0.046	78.7 ± 5.2 (3C) −13.4 ± 2 (8H)	0.99
L6 (8H)	1.811 ± 0.04	0.978 ± 0.133	82.2 ± 3.6	0.99

First we have analyzed the sample area (ROIs coded B1 and B2) surrounding the defect rich sample surface. For the case of the polar plots corresponding to ROIs B1 and B2 (Fig. 2c), the fitting with Eq. (2) provided values for $-{\chi }_{33}^{(2)}/{\chi }_{15}^{(2)}$ of 1.606 ± 0.052 and 1.628 ± 0.06, respectively which are in good agreement with the expected theoretical value of 1.6 for 4H-SiC^31^. An unexpected result, if Kleinman’s condition holds (${\chi }_{31}^{(2)}={\chi }_{15}^{(2)}$) is obtained in the case of B2. While for B1 ${\chi }_{31}^{(2)}/{\chi }_{15}^{(2)}$= 0.992 ± 0.084 which is in agreement with Kleinman’s condition, for B2 we have obtained ${\chi }_{31}^{(2)}/{\chi }_{15}^{(2)}$= 1.344 ± 0.067. A much lower quality of fitting (R² < 0.45) was obtained in this case when the value of ${\chi }_{31}^{(2)}/{\chi }_{15}^{(2)}$ was kept fixed to unity. Because the $-{\chi }_{33}^{(2)}/{\chi }_{15}^{(2)}$ distribution is centered at 1.628, which is close to the theoretical value of 1.6, we are inclined to believe that the divergence from the expected value of 1 for ${\chi }_{31}^{(2)}/{\chi }_{15}^{(2)}$ might be due to experimental errors.

The SHG intensity dependence for the area surrounding the SFs (ROIs B1 and B2) confirmed the 4H-SiC polytype for the epilayer but also allowed us to propose a possible explanation for the existence of these SFs. A considerable difference can be observed between the orientation angle computed by fitting the experimental data with Eq. (2) φ = 29° ± 3.4° for B1 and φ = 76.8° ± 5.2° for B2, respectively. This difference suggests the presence of a grain boundary which in some cases has been shown to be the origin of SFs^32^.

In the following we show that by analyzing the PSHG data for the ROIs L1 to L6 which are marked in Fig. 2a an image contrast absent in the intensity-only SHG image (Fig. 2a) can be used to demonstrate three distinct SHG responses and further to discriminate between different SiC SFs.

The SHG intensity corresponding to L2 and L4 exhibits a four-fold symmetric response as a function of the incident laser beam polarization. In the case of bulk 3C-SiC, a twofold rotational symmetry appears in components of the second-order susceptibility tensor. According to Neumann’s principle, this twofold symmetry shows up also in the SH electric field, resulting in a fourfold symmetry in the SHG intensity^23^. When fitting the experimental data for these two areas with Eq. (1) a good fitting for L2 (R² > 0.9) was obtained, while for L4 the results showed a much lower quality of fitting (R² < 0.75). This led to the conclusion that L2 features are made of fourfold structures and are quite similar to the well-known SHG signatures of 3C-SiC SFs in 4H-SiC. On the other hand, when fitting the experimental data on L4 with a linear combination of Eqs (1) and (2) better results were obtained (R² > 0.95), suggesting that this line might be a combination between cubic and hexagonal polytype SFs. The parameters retrieved for the hexagonal structure are $-{\chi }_{33}^{(2)}/{\chi }_{15}^{(2)}$= 1.93 ± 0.026, which is close to the theoretical value of 1.9^31^ for 8H-SiC, while the ratio ${\chi }_{31}^{(2)}/{\chi }_{15}^{(2)}$ was close to unity (Table 1).

The PSHG response seen as a linear combination between a cubic and a hexagonal response can be explained by considering that the dimension of individual SFs is much smaller than the optical resolution and that in this situation SFs cannot be individually resolved. In this case the PSHG response would be an average over the beam diameter of the individual SFs’ SHG responses. Hence, for closely packed 3C and 8H SFs the PSHG response would be a combination of these two, with a higher proportion for the dominating SFs.

For L3 and L6 the experimental data were fitted with Eq. (2) retrieving a ratio ${\chi }_{31}^{(2)}/{\chi }_{15}^{(2)}$ close to unity and $-{\chi }_{33}^{(2)}/{\chi }_{15}^{(2)}\,$≈ 1.82. The good quality of fitting (R² > 0.95) indicates that these features in the images are of hexagonal structure, while the value of $-{\chi }_{33}^{(2)}/{\chi }_{15}^{(2)}$ which is close to the theoretical value of 1.9 for 8H-SiC indicates the presence of 8H-SiC polytype SFs. The deviation from the theoretical value for 8H-SiC might be explained by the limited resolution of optical microscopy as compared with the size of the SFs. Hence, the PSHG response is an average over the surrounding area; in this particular case L3 being in close proximity with a 3C area, while L6 with a 4H area led to the decrease of the effective $-{\chi }_{33}^{(2)}/{\chi }_{15}^{(2)}$ computed by the fitting with Eq. 2.

For L1 and L5 fitting the experimental data with a linear combination of Eqs. (1) and (2) retrieved $-{\chi }_{33}^{(2)}/{\chi }_{15}^{(2)}$ close to the theoretical value of 1.9 for 8H-SiC and ${\chi }_{31}^{(2)}/{\chi }_{15}^{(2)}$ close to unity for the hexagonal structure. Even though for L1, L4 and L5 a combination of 3C and 8H-SiC structures was computed by the fitting procedure, a two-fold symmetry characteristic for the hexagonal structures is visible in the polar plots of L1 and L5, while for L4 a four-fold symmetry characteristic for a cubic structure is evident. This can be explained by the relative ratios of the hexagonal and cubic components in the fitting of the three data series. While for both L1 and L5 a cubic-to-hexagonal ratio of ~1.45 was estimated, for L4 a higher ratio of ~3.9 was obtained. Even though this values are only estimation of the real ratio, they indicate that for the case of L4 the cubic structure has a more pronounced effect over the hexagonal structure in the PSHG response.

**Figure 3:**
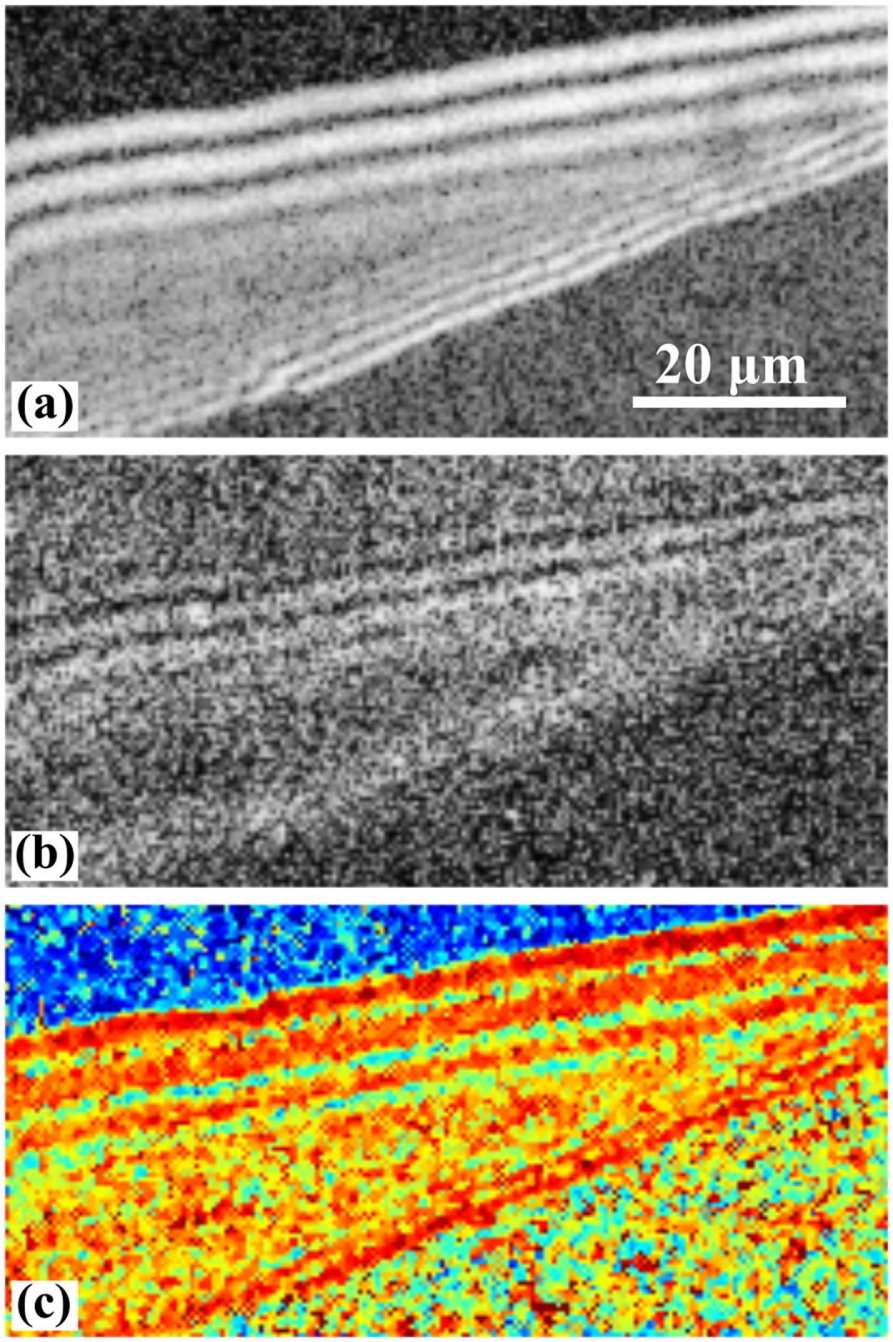
Anisotropy evaluation by using β computed on the same sample area as in Fig. 2. (**a**) SHG image acquired with the analyzer positioned parallel with the incident laser beam polarization. (**b**) SHG image acquired with the analyzer positioned perpendicular with the incident laser beam polarization. Histogram equalization^33, 34^ is used for both images because the values in SHG intensity in the case of the analyzer positioned perpendicular to the incident polarization renders pixel values that obstruct the visualization. (**c**) Anisotropy map indicating the β values for each pixel in the image.

By placing an analyzer in front of the detector the parallel and perpendicular SHG components were acquired for the calculation of the anisotropy factor (see Methods section). The anisotropy factor was computed from each set of images corresponding to parallel and perpendicular SHG components on a pixel-by-pixel basis using Eq. 3 and the values for each pixel are displayed as a color-coded map, where the β values are represented from blue to red (Fig. 3).

**Figure 4:**
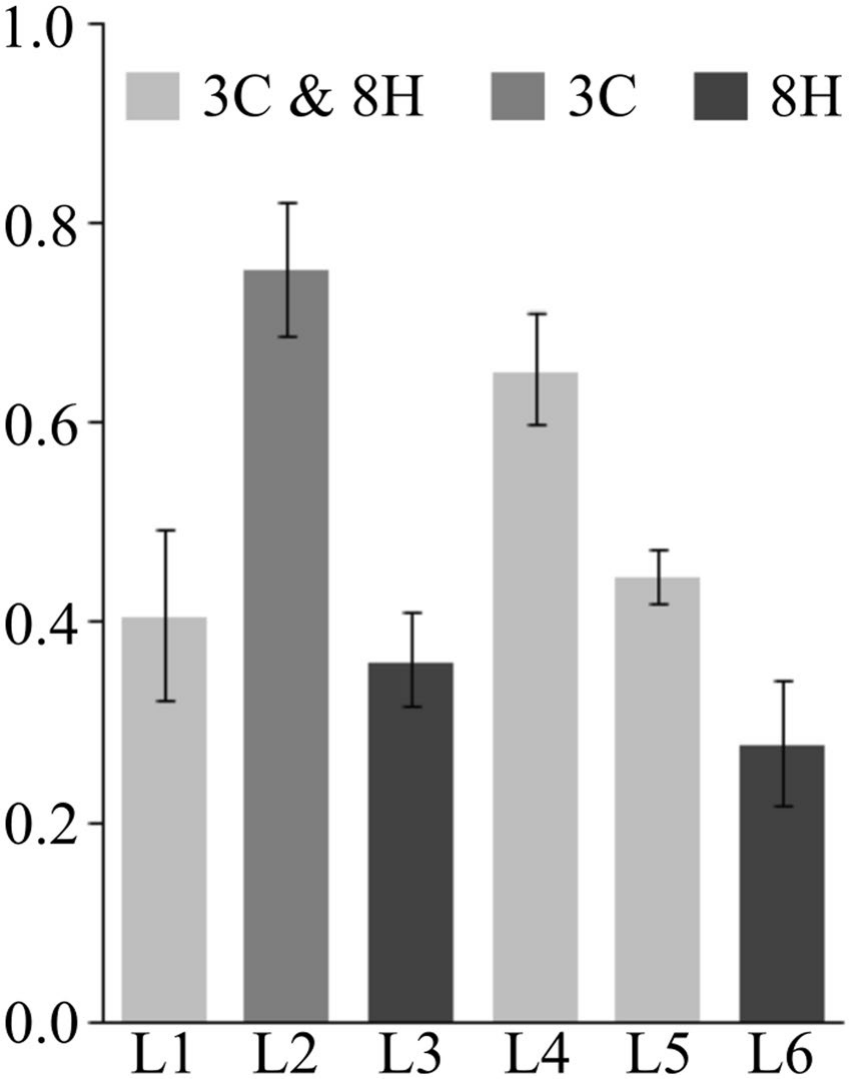
Average anisotropy factor (β) calculated for the ROIs in Fig. 2a with bars indicating the 99% confidence intervals. Based on non-overlapping confidence intervals, significant differences can be seen between the identified 3C and 8H SFs. However, the difference between 3C & 8H and 8H (more precisely between ROIs L1, L3 and L6) is not statistically significant.

By analyzing the anisotropy data for ROIs L1 to L6 (Fig. 3c), we show that the average value of β (Fig. 4) can be used to discriminate between different SiC SFs. We have previously identified three distinct behaviors by fitting the SHG intensity dependence on the laser beam polarization with either Eq. 1, Eq. 2 or a linear combination of the two.

An increase in the β value is evident when moving from 8H SFs to 3C SFs. In between these values we find the β values for the ROIs with a combined 3C and 8H behavior with L1 and L5 closer to the β value for 8H and L4 closer to the β value for 3C. These results are consistent with the previous estimation-based statement that ROIs L1 and L5 present a more pronounced 8H behavior and hence more hexagonal SFs might exist in these ROIs while L4 contains more 3C SFs.

Based on a paired student’s t-test between the mean values of β for each pair of ROIs, the difference in the mean values of β is statistically insignificant for only two cases. When comparing L3 with L6 (p > 0.05) and L1 with L5 (p > 0.3), respectively, the statistically insignificant difference can be explained by the fact that both ROIs were *apriori* identified as 8H SFs, and 3C & 8H areas respectively. On the other hand, for the comparison of L1 and L3 (p > 0.27), statistically there are not convincing evidence that they differ, hence β can not be used to differentiate between these ROIs.

In the case of all areas for which the SHG intensity dependence on the laser beam polarization was fitted with the theoretical curves, the observed deviations from the model which were not quantified by Δ (see Methods section) were generated by the depolarization of the incident laser beam. Even though we have used a quarter-wave plate to compensate for the ellipticity introduced by the microscope and a relatively low NA objective, a more precise control of the incident polarization is required.

## Discussions

Both the SHG intensity dependence and the SHG anisotropy analysis provide a differentiation between 3C and 8H SFs in 4H SiC epilayers. The advantage of the β evaluation over SHG intensity fitting is the processing time. The former method only needs the acquisition of two images with orthogonal polarizations and the computing of β maps. This analysis provided inconclusive results for areas with different polytypes (e.g. the case of L1 and L3), which the diffraction-limited optical SHG microscopy can’t resolve. On the other hand, the SHG intensity fitting using theoretical curves is slower, because it requires the acquisition of more linear polarization dependent images for an accurate fitting. As an advantage over the previous method, although it uses the same diffraction-limited images as the β evaluation, we have showed that the SHG intensity fitting can indicate areas with combined polytypes behavior.

A limitation of these methods is connected to the inherent diffraction limited resolution of PSHG microscopy. In the case of our experiment, maximum achievable resolution was 600 nm. At this scale SFs characterization cannot be done at an individual level. However, this limitation can be alleviated by employing image processing methods for optical resolution enhancement^35, 36^. Moreover, the proposed methodology can be adapted to SHG scanning near-field optical microscopy^37^, an investigation technique capable of nanoscale resolutions.

In conclusion, we have demonstrated a noninvasive optical microscopy technique based on polarization-resolved second harmonic generation for determining the polytype structure within silicon carbide stacking faults. By combining SHG intensity dependence on the laser beam polarization with anisotropy factor mapping, this technique can extend the “optical signature” concept as an all-optical set of investigation methods for the characterization of stacking faults in silicon carbide, which is fast and non-destructive. We have shown that PSHG microscopy enhances the “optical signature” in the case of SFs by its increased sensitivity to different SiC polytypes, being able to differentiate between hexagonal (8H) and cubic (3C) stacking faults in a 4H-SiC sample. Because the β evaluation offers a fast mapping of the SFs and both methods are non-invasive they can be used for the in-line evaluation of SiC production process. This could be of interest, for example, in the quality control of SiC epilayers.

## Methods

### Experimental setup

**Figure 5:**
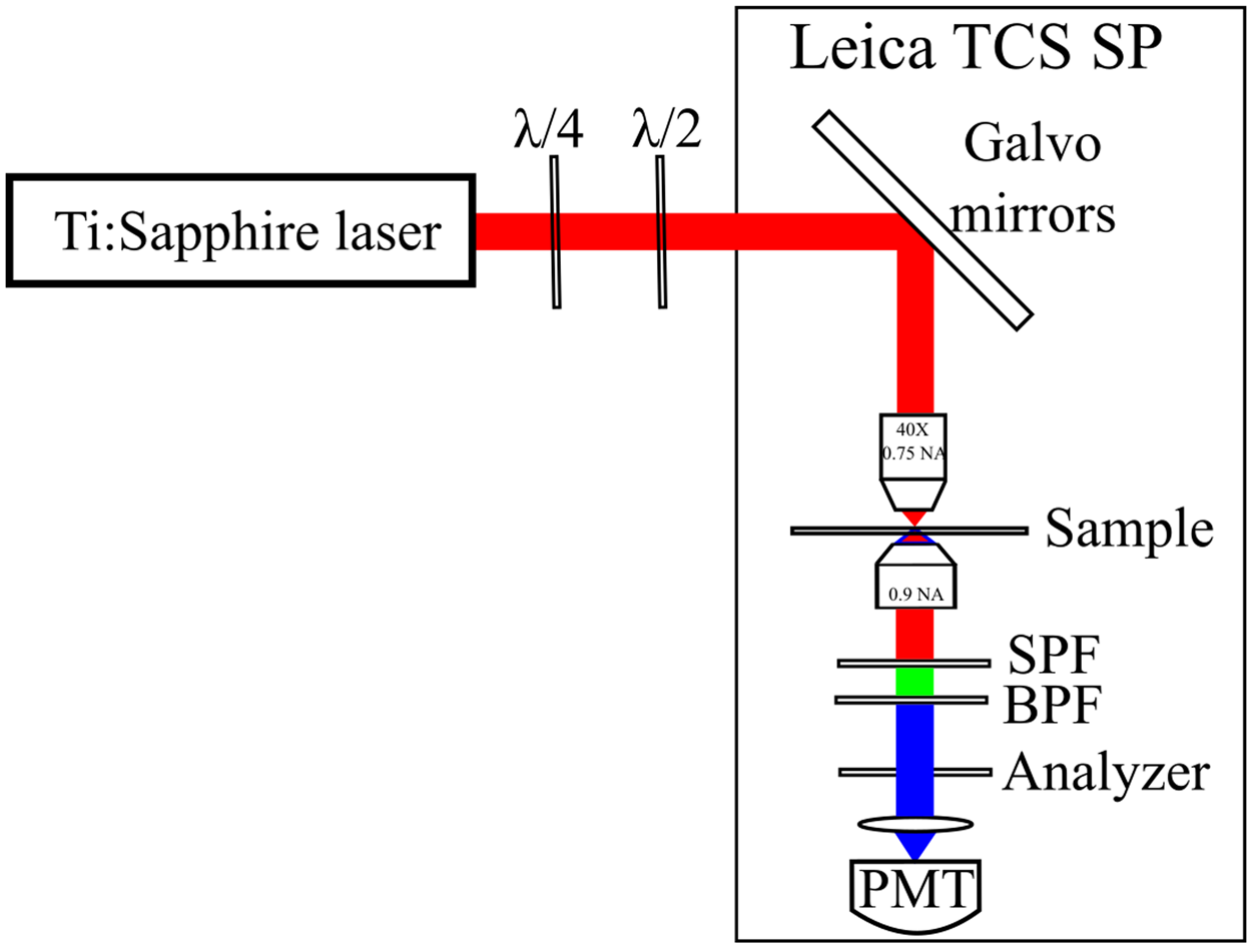
Polarization-resolved SHG imaging setup. λ/4 – quarter-wave plate; λ/2 – half-wave plate; SPF – short-pass filter; BPF – band-pass filter; PMT – photomultiplier tube.

In the reported experiment SHG imaging was performed using a Leica TCS SP confocal laser scanning microscope in a transmission geometry using normal incidence excitation (Fig. 5). The pump radiation was supplied by a mode-locked Ti:Sapphire laser (Tsunami, Spectra Physics) operating at 80 MHz repetition rate, with 150 fs duration pulses and centered at a wavelength of 780 nm. The laser beam was focused on the sample surface by using a microscope objective with a magnification of 40X and a numerical aperture (NA) of 0.75. The SHG signal was collected in the forward direction by using a 0.9 NA condenser lens, filtered by a combination of a short-pass filter (FF01-750/SP-25, Semrock) and a band-pass filter (FB390-10, Thorlabs) and directed to the transmission photomultiplier tube (PMT). An analyzer was used to select the polarization components of the SHG radiation lying either parallel or perpendicular to the polarization of the excitation laser beam. The SHG character of the detected radiation was verified by its quadratic power dependence on the excitation laser beam intensity. In our setup, the fundamental beam is normal incident (along *z* axis) and is linearly polarized in the (*x*, *y*) image plane with α the angle of polarization considered as shown in the inset in Fig. 1b. We could freely rotate the incident laser beam polarization to obtain the SHG intensity dependence by a combination between an achromatic quarter-wave plate (AQWP05M-980, Thorlabs) and an achromatic half-wave plate (AHWP05M-980, Thorlabs) located before the microscope input port.

### Test samples

We have used a 4H-SiC epitaxial layer with a thickness of 18 μm which was grown by chemical vapour deposition (CVD) on a 4H-SiC substrate in a horizontal resistively heated hot wall CVD reactor^38^. Having a bandgap of 3.26 eV, both the 4H-SiC epilayer and the substrate are transparent to both the incident laser beam and the corresponding SHG wavelength (390 nm) allowing for volume investigation and in a transmission configuration.

### Image analysis

In order to identify different SFs in the 4H-SiC sample and in particular to differentiate between 3C and 8H SFs, intensity PSHG images were fitted with theoretical curves. Regions of interest (ROIs) were selected and coded as L1 to L6 for the case of alternating high and low SHG intensity lines, while B1 and B2 are defect free regions which are expected to reveal a 4H-SiC behavior, consistent with the sample epilayer. For the case of the cubic SFs, we have considered that 3C-SiC has a zinc-blende structure corresponding to the $\bar{4}3\,m$ crystal class^39^, while the hexagonal polytypes correspond to the *6* *mm* crystal structure^31^. Under the consideration that the nonvanishing components of the second-order nonlinear susceptibility tensor are reduced to only ${\chi }_{14}^{(2)}$ and ${\chi }_{13}^{(2)}$, ${\chi }_{15}^{(2)}$, ${\chi }_{33}^{(2)}$ for the case of 3C-SiC and hexagonal SiC polytypes, respectively‚ the standard laser beam polarization dependence of the SHG intensity is given by:

1 $${I}_{3C} \sim {\chi }_{14}^{{(2)}^{2}}\,{\sin }^{2}\,2(\phi -\alpha )+{\rm{\Delta }}$$

2 $${I}_{H} \sim {\sin }^{2}\,2(\varphi -\alpha )+{(\frac{{\chi }_{31}^{(2)}}{{\chi }_{15}^{(2)}}{\sin }^{2}(\phi -\alpha )+\frac{{\chi }_{33}^{(2)}}{{\chi }_{15}^{(2)}}{\cos }^{2}(\phi -\alpha ))}^{2}+{\rm{\Delta }}$$

where α is the incident laser beam polarization angle, while φ is the orientation angle of the six-fold symmetry axis of the hexagonal crystal structure as depicted in the inset of Fig. 1b.

In this way, information on the crystal orientation and the nonlinear susceptibility components can be extracted. Differentiation between hexagonal SiC polytypes was made by considering that the $-{\chi }_{31}^{(2)}/{\chi }_{15}^{(2)}$ ratio decreases with hexagonality^31^, varying from 2 for the cubic polytype to 0.6 for the purely hexagonal 2*H* polytype. Experimental data were fitted with Equations 1 and 2, and the quality of the fitting procedure was quantified by the coefficient of determination R² (0 < R² < 1), a value closer to unity indicating a better fitting. Good fitting with Eq. 1 demonstrates a 3C behavior, while a good fitting with Eg. 2 demonstrates a 4H behavior. If the previous two cases offered poor results (R² < 0.8) a linear combination of the two equations was used. All values are given as mean plus or minus the standard deviation. The parameter Δ was added to include both experimental errors and any deviations from the theoretical model^40^.

For a fixed incident laser beam polarization a set of two images at orthogonal analyzer positions was collected and the SHG anisotropy factor was calculated by^29^:

3 $$\beta =\frac{{I}_{\parallel }-{I}_{\perp }}{{I}_{\parallel }+2{I}_{\perp }}$$

where ${I}_{\parallel }$ and ${I}_{\perp }$ are the SHG intensity detected by using an analyzer parallel and perpendicular to the laser beam polarization, respectively.

According to Eq. (3) anisotropy maps were computed for each set of images acquired at orthogonal analyzer positions and an average *β* value was further used to differentiate between 3C or 8H SFs.
